# Rethinking Air Quality and Climate Change after COVID-19

**DOI:** 10.3390/ijerph17145167

**Published:** 2020-07-17

**Authors:** Joseph Ching, Mizuo Kajino

**Affiliations:** 1 Meteorological Research Institute, 1-1 Nagamine, Tsukuba, Ibaraki 305-0052, Japan; kajino@mri-jma.go.jp; 2 Faculty of Life and Environmental Sciences, University of Tsukuba, 1-1-1 Tennodai, Tsukuba, Ibaraki 305-8572, Japan

**Keywords:** COVID-19, airborne transmission, climate change, air quality, environmental public health, the Anthropocene

## Abstract

The world is currently shadowed by the pandemic of COVID-19. Confirmed cases and the death toll has reached more than 12 million and more than 550,000 respectively as of 10 July 2020. In the unsettling pandemic of COVID-19, the whole Earth has been on an unprecedented lockdown. Social distancing among people, interrupted international and domestic air traffic and suspended industrial productions and economic activities have various far-reaching and undetermined implications on air quality and the climate system. Improvement in air quality has been reported in many cities during lockdown, while the death rate of COVID-19 has been found to be higher in more polluted cities. The relationship between the spread of the SARS-CoV-2 virus and air quality is under investigation. In addition, the battle against COVID-19 could bring short-lived and long-lasting and positive and negative impacts to the warming climate. The impacts on the climate system and the role of the climate in modulating the COVID-19 pandemic are the foci of scientific inquiry. The intertwined relationship among environment, climate change and public health is exemplified in the pandemic of COVID-19. Further investigation of the relationship is imperative in the Anthropocene, in particular, in enhancing disaster preparedness. This short article intends to give an up-to-date glimpse of the pandemic from air quality and climate perspectives and calls for a follow-up discussion.

## 1. Introduction

The world has been in a panic caused by the pandemic of COVID-19 since the beginning of 2020 [1]. At the moment of composing this article, the unsettling numbers of confirmed cases and the death toll worldwide due to COVID-19 have reached over 12 million and more than 550,000 respectively, according to the global database [2]. Currently, all countries have been busy in curbing the pandemic. To adopt the strategy of “flattening the curve”, the whole Earth has been on an unprecedented lockdown along with various measures from personal to national levels. These measures have impacted on climate and air quality, whereas the environment and climate in return could affect the spread of SARS-CoV-2, the virus leading to COVID-19. The battle against the pandemic of COVID-19 is characterized by human intervention. The intertwined relationship among environment, climate change and public health [3,4,5] is exemplified in the pandemic of COVID-19. Further investigation of the relationship is imperative in enhancing disaster prevention and preparedness, in particular in the Anthropocene [6,7,8]. While it is not possible to give a comprehensive review at this moment due to a large volume of ongoing studies in many aspects, this brief review intends to give an up-to-date glimpse of the pandemic from air quality and climate perspectives, and by raising some critical questions to initiate follow-up discussion and inspire further research.

## 2. Airborne Transmission of COVID-19

It is vital to unravel and then acknowledge the channels of transmission of infectious diseases in order to design infectious control measures and COVID-19 is no exception. In earlier times, close contact and virus-contaminated surfaces (fomite) [9] were believed to be the principle transmission channels of the SARS-CoV-2 virus. Gradually, an increasing amount of scientific evidence has gravitated to support possible airborne transmission of the SARS-CoV-2 virus [10,11,12,13,14], albeit a controversy at the moment of compiling this article ([15] and references therein). Aerosol particles were found to play a critical role in the airborne transmission of the SARS-CoV-2 virus [9,16,17]. Particles carrying the virus originated from patients could be expelled through exhalation, talking, sneezing and coughing [18,19]. The expelled particles travel in ambient environments to different spatial extents and have an atmospheric lifetime depending on (1) particles sizes, (2) indoor ventilation or outdoor air movement and (3) ambient temperature and humidity [20,21,22,23,24,25,26]. Considering the evaporation and falling of the droplets indoors, Xie and co-authors determined that particles with sizes between 60 μm and 100 μm would fully evaporate before falling for 2 m, subject to environmental conditions [20]. According to Bourouiba [12], in the occasion of forced discharge, the turbulence cloud carrying the virus-containing particles could possibly reach as far as 6 m to 8 m. Prather, Wang and Schooley [17] suggested that universal mask wearing is an effective way to minimize the spread of the virus among persons amid a good few asymptomatic patients.

**Figure 1:**
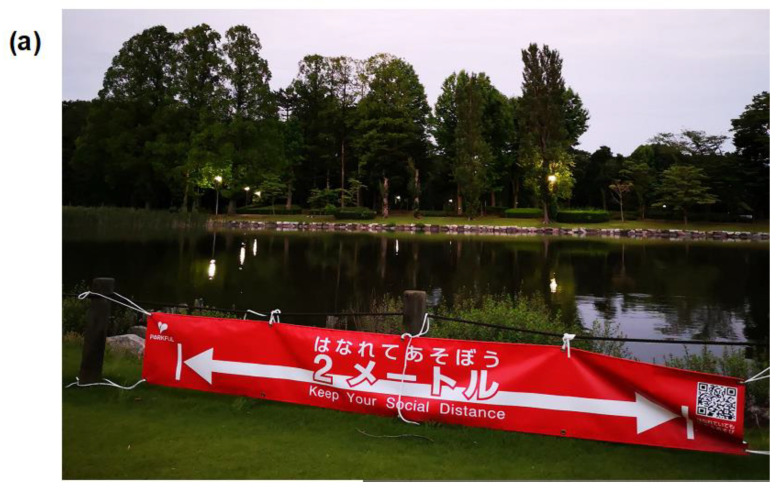

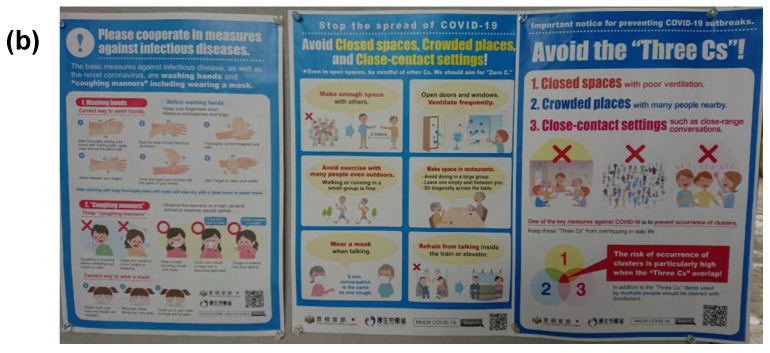
(**a**) A banner reminding park goers to keep a social distance of 2 m. (**b**) Posters on a public notice board showing the preventive measures recommended by Japan Ministry of Health, Labour and Welfare [36].

Tackling the COVID-19 pandemic is an interdisciplinary mission that involves natural science, medical science and social science [27]. Within the field of natural science, apart from climate scientists and environmental scientists, aerosol scientists have a particular role in answering questions about the airborne transmission of the SARS-CoV-2 virus [11]. Specific questions include, but are not limited to, (1) the lifetime and the fate of the virus carrying aerosol particles in both indoor and outdoor environments [28] and (2) the interactions between the virus and other chemical compositions within particles [29,30]. The answers to these questions combined with relevant researches from virologists and epidemiologists could elucidate the physiological and environmental factors determining contagiousness of the SARS-CoV-2 virus. Results of these interdisciplinary studies are essential in (1) the detection of the virus-containing aerosol in both indoor and outdoor environments [31,32,33], (2) the development of infection control measures in both indoor and outdoor environments [28,34,35] and (3) the recommendation to the general public for infection prevention (Figure 1).

## 3. Air Quality and COVID-19

Aerosol particles, also known as PM_2.5_ (particles with a diameter less than or equal to 2.5 micrometers), have been accused of deteriorating air quality and leading to various kinds of pulmonary and cardiovascular diseases causing premature death [37]. A few studies have indicated that cities with poorer air quality are prone to a higher death rate of COVID-19 than cleaner ones [38,39,40]. The mechanisms behind the positive correlation remain to be explored further [41,42,43,44]. It is reasonable to speculate and investigate the roles of ambient aerosol particles in (1) promoting airborne transmission of SARS-CoV-2 in both indoor and outdoor environments and (2) promoting a higher premature death rate of COVID-19. Furthermore, for (2), it is worth examining whether the higher premature death rate is due to the weakening of immunity from the underlying chronic diseases caused by poor air quality or due to the interactions between the SARS-CoV-2 virus contained in the particles and other coexisting chemical components.

Since March 2020, many cities have been on lockdown. A few studies reported significantly improved air quality and visibility in many cities and postulated or attributed that to the reduced emissions from industrial productions, local transport, power generations and many other economic activities. This is evident from satellite images and ground-based measurements of PM_2_._5_ and major gaseous pollutants in many cities [45,46,47,48,49,50]. Measurements of NO_2_ showed that extensive reductions of an average of 53% happened in urban areas in Europe due to restrictions on transport, and the reduction of NO_2_ in Wuhan, China amounted to 57% with respect to the 3-year average level (2017–2019). Urban PM_2.5_ concentrations were found to decrease by about 8% and 42% in Europe and Wuhan, respectively [51]. However, the daily average ozone concentrations in urban centers increased in a number of European cities as well as Wuhan. The increase during the lockdown ranged from 2.4% to 27% in European cities and 36% in Wuhan. The reduction of NO_2_ was blamed for the increase in ozone concentrations [51]. Bekbulat and colleagues calculated the temporally-corrected time series of ozone and PM_2.5_ from US EPA daily data and concluded that both PM_2.5_ and ozone were higher than their corresponding expected values in the post-COVID-19 period across the US [52]. In addition, a number of studies revealed generally that there has been a decrease in NO_2_ and PM_2.5_ and an increase in ozone concentrations during or after the lockdown in Barcelona, Spain [53], Delhi, India [46] and cities in Central China [54]. In the UK during April, although NO_2_ concentrations were found to be lower with respect to the 4-year average (2015–2019), PM_2.5_ and ozone did not show any significant difference [55]. It has been evident from the observations in various countries that air quality improvement is achievable. However, it should also be acknowledged that variations exist among individual countries in terms of magnitudes and temporal patterns of the changes in the air quality induced by their respective lockdown policies. These variations are due to the differences in upwind regions, topography, meteorological characteristics and emission characteristics, which are related to socioeconomically-driven human activities. To devise a city- or nation-specific clear air policy, the lockdown has offered an evaluation testbed of emission control policy in mitigating severe air pollution facing humankind globally [56,57,58].

Apart from the outdoor air quality, indoor air quality has been a concern regarding the spread of the disease and the associated preventive and infection control measures. Places of special interest include, but are not limited to, toilets, hospitals, clinics, aircrafts and train compartments. Recent laboratory studies [59,60] revealed the presence of SAR-CoV-2 viral RNA in patients’ feces. Comparing the presence of viral RNA in respiratory swabs (16.7 days in their sampled patients), that in fecal samples lasted for a longer period (27.9 days) with respect to the first symptom onset [58]. Along with that, it has been well recognized that a toilet plume composed of virus containing bioaerosol particles could be produced during flushing [61,62]. Johnson and co-authors found that 95% of toilet flushing generated droplets were less than 2 μm and >99% less than 5 μm in diameter and the amount of airborne bioaerosols increased with flush energy [61]. Measurements by Knowlton and colleagues showed the particles’ diameter peaked at 0.3 μm and stayed in the air for longer than 30 min after the flush. The bioaerosols generated by toilet flushing contained live pathogens and, apart from lingering in the air, could contaminate surfaces [62]. These findings raise an essential question pertinent to infection control measures carried out in highly contagious places such as hospitals, which is “Is flushing toilet a possible mean of SARS-CoV-2 transmission through the generation of an aerosol plume?” McDermott and colleagues further mentioned a few research questions regarding the potential roles of bioaerosols from toilet flushing in transmitting SARS-CoV-2 [63]. Meanwhile, putting a lid down before flushing is a simple precaution to reduce the amount of airborne bioaerosols.

From indoor air quality and microclimate perspectives, several venues should be given particular considerations, namely (1) processes generating clouds of virus laden aerosols, such as the use of nebulizers during medical treatment and flushing toilets, (2) air exchange frequency and ventilation strategy between the outdoor and indoor environments, (3) the cleanliness of the filters of air conditioning systems, (4) temperature and relative humidity control strategies and (5) the density and flow of humans indoors. All of these are expected to impact on the duration of the bioaerosols remaining airborne, the extent of contamination of the nearby surfaces and the viability of the SARS-CoV-2 virus contained in the particles.

## 4. Climate, Climate Change, COVID-19 and Beyond

While the cleaner air during lockdown has demonstrated that it is feasible for human society to improve air quality, the climate implications of reduced aerosol and gaseous pollutants emissions during the global pandemic of COVID-19 remain to be scrutinized. On the other hand, the climate could modulate the spread of the pandemic and climate change could lead to the emergence of novel infectious diseases.

Decarbonization has been suggested by climate scientists and environmental protection activists as a solution to global climate change. Since the emission reduction during COVID-19 lockdown is of a global scale, it could offer some clues about the efficacy of greenhouse gases and PM_2.5_ reductions of a hypothetical global scale decarbonization. A recent preliminary calculation showed a 17% cut of daily global carbon dioxide emissions in April 2020 compared with the 2019 average [64]. This global scale lockdown is analogous to a natural testbed of a geoengineering experiment offered by the volcanic eruption of Mount Pinatubo in the Philippines in 1991 [65,66,67,68]. Another venue for climate-relevant evaluation is air traffic. Since the outbreak of COVID-19, both international and domestic air traffic has been diminishing. The impacts of air traffic on the climate can be explored by contrasting the global climate model simulated radiative forcing due to air traffic [69,70] before COVID-19 and during COVID-19 lockdown.

Although it has been reported that the air quality has improved in many cities since lockdown, an ad hoc emission reduction of this kind undoubtedly does not offer a fundamental and long-lasting solution to air quality deterioration and climate change. The emission of PM_2.5_ and gaseous pollutants will likely bounce back when economic activities resume. In addition, during COVID-19, an enormous amount of one-time disposable items, for example, surgical masks, personal protection equipment and commodity packaging, have been discarded [71,72]. This waste treatment potentially results in an extra amount of air pollutants.

The outbreak of influenza and SARS [73] in 2003 (caused by SARS-CoV-1) demonstrated clear seasonality and latitudinal dependence [74,75,76,77]. Some studies suggested that the temperature and humidity of a locality influenced the virus spread [24,25,78,79,80,81,82]. In light of the seasonal emergence pattern of SARS-CoV-1, it has been speculated that high temperatures and high humidity may suppress the transmission of SARS-CoV-2. It was believed that COVID-19 could follow a seasonal pattern alternating between the southern and the northern hemisphere, characterized by an outbreak in the winter-spring, then a decline in the summer and followed by a recurrence in the next winter. However, enormous locally infected cases found in countries having high ambient temperatures such as Singapore, Indonesia and Malaysia put the speculation into question [83]. It is possible that the role of climate in the transmission of COVID-19 could be modulated by other factors including population density, age distribution, hygiene level, policy intervention and local culture that are specific to individual countries. This probably gives rise to the ambiguous seasonal signal of local SARS-CoV-2 transmission, if any. Further studies are needed to elucidate whether the climate plays any role and to what extent in regulating the transmission of SARS-CoV-2. This could offer some hints for an early warning of the next wave of an outbreak. In the long term, the temperature, humidity and latitudinal dependence of some infectious diseases implies that climate change could further complicate the future spread of infectious diseases including COVID-19. Besides, several studies have indicated the enhanced incidence of infectious diseases amid a warming climate through changing precipitation patterns and temperature variations [84,85,86,87]. More frequent occurrences of extreme weather could contribute in this regard. In addition, it is worrisome that under global warming, ancient micro-organisms, including viruses, bacteria and fungi that have been locked in permafrost, icebergs and glaciers, could be released into the atmosphere upon the melting of the ice [88,89,90]. This poses a potential threat to humankind since the immune system of the modern human has never encountered those pathogens carried by the ancient micro-organisms [90].

## 5. Looking Forward

Fighting against COVID-19 has provided humankind an enlightening lesson, which has established a database and experience for developing future strategies to combat global pandemics, improve air quality and mitigate climate change. In the battle against the pandemic, it is obvious that the combined effort of academic institutions, medical institutions, private corporates, governments and general public is vital in order to bring the pandemic under control. This is also applicable in global air quality improvement and climate change mitigation. Particularly, academic researchers of relevant areas should participate in providing advice to policy makers in formulating science-based decisions, assessing risk and issuing early warning and contribute to raising general public awareness through education and communication. Besides, since the success in restricting the emission of CFCs [91] in healing the ozone hole over Antarctica, international efforts such as the recent Paris Agreement have resulted in no significant achievement towards reducing greenhouse gas emissions. International collaboration is treasured not only in fighting against COVID-19 but also in global warming mitigation and air quality improvement.

The era of Anthropocene [6,7,8] is characterized by the nexus of the environment-climate-public health-society [3,4,92]. Human activities affect the surrounding environment and the Earth’s climate system significantly and conversely the environmental and climate changes present extreme weather, natural disasters and threats to public health. Within the context of global warming, increasingly frequent extreme weather could enhance the emergence and spread of various kinds of infectious diseases. Close monitoring, effective international communication and early warning systems of global pandemics are desired. In recent decades, when sustainable development goals (SDGs) have been emphasized [93,94], the societal prevention and preparedness towards natural and manmade disasters [95] as well as adaptation and resilience to the environmental and climate changes have become increasingly indispensable. Shifting to a green economy may be a solution to global sustainability.

## 6. Conclusions

We conclude this brief communication by summarizing the unresolved issues discussed and raising corresponding follow-up research questions in Table 1. Table 2 lists a few important research questions for post-COVID-19. We believe ongoing scientific research will continue to unlock how the pandemic of COVID-19 interacts with the fabric of society. What is as important as scientific research is how individuals respond to the challenges in all facets as a responsible global citizen. Perhaps everyone, not only scientists, should reflect on the questions “What have we learnt from the global pandemic of COVID-19?” and “Are we prepared for the challenges ahead, if this time is only a rehearsal?”.

## Figures and Tables

**Table 1:** Some of the unresolved issues about COVID-19 and corresponding follow-up questions suggested in the article.

Unresolved Issues	Follow-Up Questions
Microphysically, there is a controversy over whether airborne transmission of SARS-CoV-2 takes place, although it is increasingly evident that it is the case.	How do the SARS-CoV-2 virus containing particles interact with other ambient aerosol particles? How does the SARS-CoV-2 virus interact with other coexisting components in the same particles? What are the factors determining the atmospheric lifetime and fate of SARS-CoV-2 virus containing particles? What is the minimum amount SARS-CoV-2 virus required to cause COVID-19? Consequently, how do those factors affect the infectiousness of the SARS-CoV-2 virus? How does the effectiveness of airborne transmission of SARS-CoV-2 virus compare with transmission through close physical contact and contaminated surfaces (also known as fomite)? How do we compile science-based infectious control measures and policies and recommendations to the general public in both indoor and outdoor public areas?
2. Macrophysically, there is a seemingly positive correlation between the COVID-19 death rate and pollution level in many cities. It is uncertain if such a positive correlation is prevalent worldwide. Also, the underlying mechanisms responsible for that remain to be explored.	
3. The impacts on the global climate from the drastically reduced greenhouse gases and particulate matter (PM) emissions during lockdown remain to be examined.	5. How does the reduced emissions during lockdown affect air quality and global climate quantitatively and qualitatively? What are the mechanisms behind those impacts? 6. How do we leverage the testbed offered by the lockdown for compiling a future emission policy for air quality improvement and global warming mitigation?
4. The impacts on air quality from the drastically reduced gaseous pollutants and PM emissions during lockdown remain to be examined.	

**Table 2:** Some of the important questions for post-COVID-19.

How would the emissions after the resumption of economics and human activities shape the air quality and climate and consequently public health?
2. How would the treatment of the extra medical and household waste (e.g., take away catering packaging) generated during COVID-19 affect the air quality and climate and consequently public health?
3. What have we learnt from the COVID-19 pandemic? Under the circumstance of potentially more frequent occurrences of infectious diseases amid climate change, are we prepared for the next?
